# Predicting visceral obesity based on facial characteristics

**DOI:** 10.1186/1472-6882-14-248

**Published:** 2014-07-16

**Authors:** Bum Ju Lee, Jong Yeol Kim

**Affiliations:** 1 Medical Research Division, Korea Institute of Oriental Medicine, 1672 Yuseongdae-ro, Yuseong-gu, Deajeon 305-811, Republic of Korea

## Abstract

**Background:**

Visceral obesity is associated with facial characteristics and chronic disease, but no studies on the best predictor of visceral obesity based on facial characteristics have been reported. The aims of the present study were to investigate the association of visceral obesity with facial characteristics, to determine the best predictor of normal waist and visceral obesity among these characteristics, and to compare the predictive power of individual and combined characteristics.

**Methods:**

Cross-sectional data were obtained from 11347 adult Korean men and women ranging from 18 to 80 years old. We examined 15 facial characteristics to identify the strongest predictor of normal and viscerally obese subjects and assessed the predictive power of the combined characteristics.

**Results:**

FD_94_194 (the distance between both inferior ear lobes) was the best indicator of the normal and viscerally obese subjects in the following groups: Men-18-50 (p ≤ 0.0001, OR = 4.610, AUC = 0.821), Men-50-80 (p ≤ 0.0001, OR = 2.624, AUC = 0.735), and Women-18-50 (p ≤ 0.0001, OR = 2.979, AUC = 0.76). In contrast, FD_43_143 (mandibular width) was the strongest predictor in Women-50-80 (p ≤ 0.0001, OR = 2.099, AUC = 0.679). In a comparison of the combined characteristics, the area under the receiver operating characteristic curve (AUC) and the kappa values of the 4 groups ranged from 0.826 to 0.702 and from 0.483 to 0.279, respectively. The model for Men-18-50 showed the strongest predictive values and the model for Women-51-80 had the lowest predictive value for both the individual and combined characteristics.

**Conclusions:**

In both men and women, the predictive power of the young and middle-age groups was better than that of the elderly groups for predicting normal waist and viscerally obese subjects for both the individual and combined characteristics. The predictive power appeared to increase slightly with the combined characteristics.

## Background

Over the past several decades, there has been a tremendous increase in the prevalence of obesity and visceral obesity (VO). VO is a major public health problem in most countries, and it is commonly related to chronic diseases, such as metabolic syndrome, diabetes mellitus, insulin resistance, and cardiovascular disease (CVD) [1-6]. Generally, VO and body mass index (BMI) are strong predictors of insulin resistance, type 2 diabetes, and CVD in both women and men [7,8]. The waist circumference (WC) measurement, which is used to diagnose VO, is a more accurate predictor of CVD or metabolic syndrome than BMI [1,2,9-12].

Over the years, a number of facial characteristic studies have attempted to characterize the association of facial characteristics with VO and have suggested that facial characteristics are strongly associated with BMI or health complications and problems [13-27]. Facial morphological characteristics can offer significant cues about genetic conditions or future health conditions [13]. For example, a study by Lee and colleagues [14] proposed a prediction method of normal weight and overweight status based on BMI using facial features and demonstrated that normal and overweight females could be classified using only facial features. A study by Sierra-Johnson and Johnson [24] simply reported the relationship of facial adiposity with VO and suggested the hypothesis that facial characteristics, such as cheek fat, are a likely alternative indicator of insulin resistance, based on previous studies of the association between facial fat and insulin resistance and metabolism. Rantala and colleagues [25] examined whether the facial and body adiposity and masculinity mediate the association of the hepatitis B antibody response with attractiveness in men. They suggested that facial attractiveness was significantly predicted by regional fat deposits in the face and was associated with the antibody response. A study by Boothroyd and colleagues [26] tested whether facial masculinity and structure could predict the past, present, and future health status of individuals and demonstrated a correlation among facial characteristics, such as rated attractiveness, rated healthiness, rated masculinity, and morphometric masculinity, with past health. The subjects with higher attractiveness showed a lower incidence of cold or influenza, and there was a correlation between facial characteristics and future health outcomes, such as healthier subjects using fewer antibiotics. A study by Sadeghianrizi and colleagues [27] showed that the skeletal structures of the faces of obese adolescents are prone to be relatively large. They suggested that bimaxillary prognathism was related to obesity and that the craniofacial morphology between obese and non-obese adolescents was significantly different. The specific elements of facial features and obesity-related diseases were well described in the study by Reither and colleagues [13].

Furthermore, facial characteristics can differ according to ethnicity, gender, and age [28,29]. A study by Zhuang and colleagues [28] examined significant differences in facial features (e.g., face shape and size) according to ethnic and gender groups. They found that the noses of African-American civilian workers were broader, shorter, and shallower than those of Caucasian workers and suggested that facial anthropometric dimensions were significantly different among ethnic groups, men and women, and age groups. Furthermore, Du and colleagues [29] suggested that the face lengths and widths of Chinese workers were shorter and wider, respectively, than those of Americans and that the nose protrusions of the Chinese workers were smaller than those of the Americans.

The purpose of the present study was to assess the association between the VO and facial characteristics of Korean adults aged 18 to 80 years, to identify the best predictor of normal waist and VO among the facial characteristics of men and women and to evaluate the predictive powers of individual predictors and combined predictors of normal waist and VO using only facial information. To answer these questions, we used statistical analysis to examine the association between VO and facial morphological characteristics in Korean adults. We also evaluated the predictive power of individual characteristics and compared the powers of the best individual predictor and the predictors based on combined characteristics to improve the prediction accuracy. An association between facial characteristics and VO was suggested in our previous study [30]. However, the results of that study were limited to only middle-aged participants, and no best predictor between normal waist and VO was found. New findings of the best predictor of normal waist and VO and the association between VO and facial features in young and middle-age groups and in the elderly group for both men and women may advance the ability to predict health problems.

## Methods

### Data collection

In this cross-sectional study, all data examined were collected from 11347 Korean men and women ranging in age from 18 to 80 years old and were acquired from the Korean Health and Genome Epidemiology study database (KHGES). Written informed consent was obtained from all subjects, and the Korea Institute of Oriental Medicine (KIOM) Institutional Review Board approved the study (No. AS10153, AJIRB-MED-SUR-12-377, and I-1210/002/002-02).

**Figure 1:**
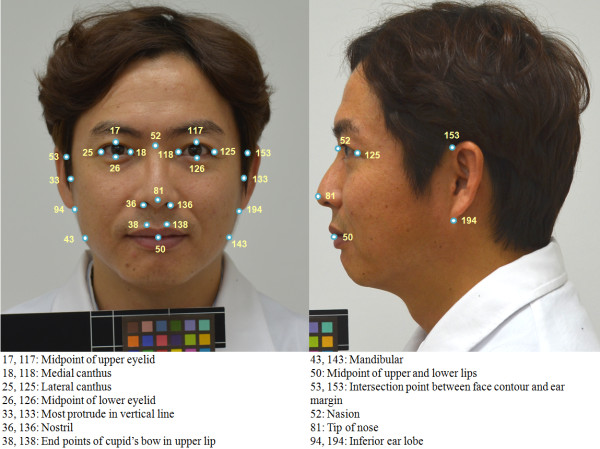
**The characteristic points on a frontal image used to extract the facial characteristics.** Photograph taken by the author.

Frontal and lateral images of the subject faces were taken with a digital camera (Nikon D5100 with an 85-mm lens; Nikon Co., Ltd., Japan) with a ruler. Clinical information, such as gender, age, height, weight, WC, and plastic surgery of the face, was recorded. Based on standardized protocols, the anthropometric characteristics were measured by well-trained observers and technicians [31,32]. The weights and heights of the subjects were measured without shoes and in light clothes. Height and weight were measured to the nearest 0.1 cm and 0.1 kg, respectively, with a digital scale (LG-150, G Tech International Co., Ltd., Republic of Korea), and WC was measured using non-elastic tape along the waist and was measured to the nearest 0.1 cm. To reduce inter-rater bias, we prepared a strict standard operating procedure (SOP) for photo-taking and WC measurement, and we observed technicians periodically. If the technicians could not produce uniform photographs or WCs according to the SOP, the technicians re-took the photographs and re-measured the WCs until they reached a certain level of accuracy. Based on identifiable points designated by doctors on the images [14,30], we extracted 15 characteristics from the frontal images. The extracted characteristics represented the horizontal or vertical distances between points n1 and n2 in the frontal image; the area of a contour formed by several points; and the ratio of 2 distances. The characteristic points on the face images are presented in Figure 1 (we obtained written consent from the subject for the use of this figure). The basic characteristics of the present study and brief descriptions are presented in Table 1.

**Table 1:** Basic characteristics of the present study

	**Men-18-50**		**Men-51-80**		**Women-18-50**		**Women-51-80**		
**Characteristic**	**Normal**	**VO**	**Normal**	**VO**	**Normal**	**VO**	**Normal**	**VO**	**Description**
Subjects	1044	514	1904	1197	1912	705	1839	2232	Number of subjects
Height	171.4 (5.849)	173.4 (5.996)	165.8 (5.699)	168 (5.625)	159.1 (5.246)	159.7 (5.239)	154 (5.633)	153.7 (5.801)	
Weight	67.42 (7.225)	82.43 (9.843)	62.83 (7.345)	74.85 (8.006)	54.28 (5.769)	66.77 (8.388)	53.14 (5.951)	62.18 (7.676)	
BMI	22.96 (2.278)	27.4 (2.7)	22.84 (2.265)	26.51 (2.33)	21.45 (2.229)	26.18 (3.091)	22.39 (2.159)	26.3 (2.856)	Body mass index
Age	37.42 (10.54)	39.74 (9.35)	60.88 (7.806)	61.22 (7.798)	38.07 (9.516)	41.13 (8.53)	59.26 (7.501)	63.08 (7.906)	
FDH_36_136	26.81 (2.959)	27.48 (2.969)	27.69 (3.766)	28.17 (3.359)	24.11 (2.47)	24.75 (3.031)	25.15 (2.501)	25.68 (2.769)	Horizontal distance between point 36 and point 136 in an image (mm)
FDV_52_50	77 (4.841)	77.57 (4.726)	77.67 (5.566)	78.41 (5.696)	72.9 (4.027)	73.21 (4.634)	73.58 (4.584)	73.19 (5.44)	Vertical distance between point 52 and 50 (mm)
FDV_52_81	47.75 (3.878)	47.75 (3.724)	47.52 (3.852)	47.66 (3.901)	45.24 (3.203)	45.02 (3.381)	44.68 (3.324)	44.16 (3.685)	Vertical distance between point 52 and 81 (mm)
FDV_81_50	29.25 (2.927)	29.82 (3.113)	30.14 (3.462)	30.75 (3.684)	27.66 (2.392)	28.19 (3.309)	28.91 (2.67)	29.03 (3.291)	Vertical distance between point 81 and 50 (mm)
FDV_38_50	9.084 (2.279)	9.357 (2.622)	8.507 (4.001)	8.532 (3.887)	9.153 (1.605)	9.3 (1.651)	8.836 (2.175)	8.677 (2.273)	Vertical distance between point 38 and 50 (mm)
FDV_138_50	9.062 (2.438)	9.309 (2.724)	8.39 (4.332)	8.449 (4.31)	9.122 (1.604)	9.256 (1.73)	8.731 (2.212)	8.618 (2.239)	Vertical distance between point 138 and 50 (mm)
FD_43_143	135.5 (9.357)	145.8 (11.3)	135.1 (10.12)	142.8 (10.25)	126.8 (8.433)	133.6 (8.537)	127.8 (8.019)	133 (9.48)	Distance between point 43 and 143 (mm)
FD_53_153	153.3 (7.935)	160.1 (8.047)	149.7 (10.05)	155.4 (9.056)	146 (6.855)	150.3 (7.439)	143.6 (7.304)	146.1 (9.094)	Distance between point 53 and 153 (mm)
FD_94_194	150.7 (8.1)	160.5 (8.045)	149.8 (9.481)	157 (9.06)	141.6 (7.004)	148.3 (7.368)	142.5 (7.098)	147 (8.604)	Distance between point 94 and 194 (mm)
FDH_33_133	156.4 (7.693)	164.5 (7.846)	154 (9.454)	160.8 (8.74)	148.2 (6.773)	153.6 (7.372)	147.1 (7.197)	150.8 (9.089)	Horizontal distance between point 33 and point 133 (mm)
FArea02	7215 (751.6)	7577 (749.5)	7084 (879.2)	7390 (827)	6475 (610.8)	6674 (644)	6352 (634.3)	6424 (831.7)	Area of the contour formed by the points 53, 153, 133, 194, 94, 33, and 53 (mm^2^)
FArea03	4225 (514.6)	4619 (576.8)	4338 (663.2)	4655 (626.5)	3747 (421.4)	4007 (499.9)	3938 (434)	4108 (664.4)	Area of the contour formed by the points 94, 194, 143, 43, and 94 (mm^2^)
FR05_psu	1.157 (0.061)	1.137 (0.153)	1.142 (0.06)	1.13 (0.085)	1.172 (0.059)	1.152 (0.056)	1.154 (0.052)	1.136 (0.052)	FDH(33,133)/FD(43,143) (mm)
FR06_psu	2.037 (0.133)	2.127 (0.143)	1.99 (0.146)	2.059 (0.165)	2.038 (0.12)	2.104 (0.143)	2.005 (0.132)	2.068 (0.146)	FDH(33,133)/FDV(52,50) (mm)
FR08_psu	1.767 (0.171)	1.887 (0.197)	1.748 (0.175)	1.832 (0.204)	1.745 (0.153)	1.833 (0.173)	1.744 (0.16)	1.826 (0.175)	FD(43,143)/FDV(52,50) (mm)
Waist	81.87 (5.458)	95.88 (5.395)	82.85 (5.322)	95.39 (4.665)	76.2 (5.514)	90.95 (5.466)	78.65 (4.773)	92.7 (6.088)	Waist circumference (cm)

VO: visceral obesity. Data are presented as the mean (standard deviation).

### Cut-off values for waist circumference and grouping

WC is appropriate for measuring visceral adiposity in a large-scale epidemiological study because it is an inexpensive, fast, safe, and easy measurement [1]. The appropriate cut-off values for WC to diagnose VO vary by ethnic group. The WC and/or BMI values of populations in Asian regions are prone to be lower than those of populations in Western regions. However, Asians tend to have risk factors for obesity-related diabetes and CVD at relatively low WC and/or BMI values [12,23]. Although VO was defined by the WHO and NCEP ATP III as a WC ≥ 90 cm for men and ≥ 80 cm for women, according to Asian-Pacific guidelines [33,34], many studies do not agree with the cut-off value of 80 cm for Korean women. These studies suggest that this cut-off point for Korean women is too low [35-37]. We adapted the recommendations of studies [35,38,39] to determine a cut-off value of WC to diagnose VO because these studies provide the latest in a series of ethnically specific cut-off points associated with the prevalence of metabolic syndrome and metabolic risk factors in Korean men and women. Therefore, the suggested cut-off values of WC for the diagnosis of VO were ≥ 85 cm in women and ≥ 90 cm in men.

To classify the age- and gender-specific groups, all data were divided into groups of 4: Women-18-50 (women aged 21–50 years), Women-51-80 (women aged 51–80 years), Men-18-50 (men aged 21–50 years), and Men-51-80 (men aged 51–80 years). Note that women who have experienced menopause show body shape changes and increased visceral adiposity [40-42] and that facial characteristics may differ according to gender and age [28,29]. The basic characteristics of the normal and VO subjects in each group are presented in Table 1.

### Statistical analysis

In all age and gender groups, the statistical analyses between the normal and VO groups were examined by binary logistic regression (IBM SPSS for Windows version 19, SPSS Inc., Chicago, IL, USA). The association of facial characteristics with visceral obesity was determined by binary logistic regression in which differences between normal subjects and viscerally obese subjects were examined after applying standardized data transformation.

Analyses of the predictive power of individual characteristics and predictions using combined characteristics were performed using binary logistic regression (Waikato Environment for Knowledge Analysis data mining software) [43]. In the prediction using combined characteristics, with the goals of building a cost-effective model, decreasing the model complexity, and improving the predictive power, wrapper-based variable selection was conducted by logistic regression and greedy search algorithm (backward elimination). For the performance evaluation, we used the area under the receiver operating characteristic curve (AUC) and Cohen’s kappa as the major evaluation criteria. Additionally, the sensitivity, 1 - specificity, precision, F-measure, and Matthews correlation coefficient (MCC) were used for the detailed performance analysis. The tests in the analysis of the predictive power using the individual characteristics and the combined characteristics were performed using 10-fold cross validation.

## Results

### Comparison of individual characteristics in the age- and gender-specific groups

The results presented in Tables 2 and 3 show the statistical analysis and predictive power of the individual characteristics in the male and female groups. The results in the Men-18-50 group (Table 2) show 13 characteristics with significant differences (p-value <0.05) between the normal and VO subjects (except for FDV_52_81 and FDV_138_50, FDV: vertical distance between two points), with 10 of these characteristics being highly significantly different (p-value < 0.0001). The strongest predictor of VO among the 15 facial characteristics is FD_94_194 (p ≤ 0.0001; OR = 4.610; AUC = 0.821, FD: distance between two points) in the Men-18-50 group. Additionally, FD_43_143 (p ≤ 0.0001; OR = 3.609; AUC = 0.788) and FDH_33_133 (p ≤ 0.0001; OR = 3.432; AUC = 0.783, FDH: horizontal distance between two points) are useful predictors. In the Men-51-80 group, differences in 10 of 12 characteristics, except for FDV_52_81, FDV_38_50, and FDV_138_50, are highly significant between normal waist and VO. The best indicator of VO in this group is FD_94_194 (p ≤ 0.0001; OR = 2.624; AUC = 0.735), which is the same as in the Men-18-50 group, but the predictive power for the characteristics in Men-51-80 is lower than that in Men-18-50.

**Table 2:** Comparison of statistical analysis and predictive power of individual characteristics in men

	**Men-18-50**			**Men-51-80**		
**Characteristic**	**p**	**OR**	**AUC**	**p**	**OR**	**AUC**
FDH_36_136	<0.0001	1.262	0.565	0.0005	1.145	0.546
FDV_52_50	0.0290	1.127	0.527	0.0004	1.142	0.541
FDV_52_81	0.9736	0.998	0.454	0.3374	1.036	0.511
FDV_81_50	0.0005	1.215	0.55	<0.0001	1.193	0.554
FDV_38_50	0.0378	1.116	0.525	0.8675	1.006	0.495
FDV_138_50	0.0743	1.098	0.522	0.7094	1.014	0.499
FD_43_143	<0.0001	3.609	0.788	<0.0001	2.460	0.722
FD_53_153	<0.0001	2.782	0.745	<0.0001	1.960	0.684
FD_94_194	<0.0001	4.610	0.821	<0.0001	2.624	0.735
FDH_33_133	<0.0001	3.432	0.783	<0.0001	2.387	0.72
FArea02	<0.0001	1.665	0.635	<0.0001	1.481	0.613
FArea03	<0.0001	2.294	0.704	<0.0001	1.850	0.662
FR05_psu	<0.0001	0.627	0.635	<0.0001	0.783	0.573
FR06_psu	<0.0001	1.942	0.685	<0.0001	1.590	0.628
FR08_psu	<0.0001	2.032	0.694	<0.0001	1.591	0.628

OR: odd ratio, AUC: area under the receiver operating characteristic curve.

**Table 3:** Comparison of statistical analysis and predictive power of individual characteristics in women

	**Women-18-50**			**Women-51-80**		
**Characteristic**	**p**	**OR**	**AUC**	**p**	**OR**	**AUC**
FDH_36_136	<0.0001	1.274	0.571	<0.0001	1.235	0.561
FDV_52_50	0.0885	1.077	0.523	0.0156	0.926	0.526
FDV_52_81	0.1246	0.934	0.515	<0.0001	0.864	0.544
FDV_81_50	<0.0001	1.235	0.558	0.1985	1.042	0.507
FDV_38_50	0.0411	1.093	0.529	0.0246	0.931	0.524
FDV_138_50	0.0652	1.083	0.524	0.1074	0.950	0.517
FD_43_143	<0.0001	2.472	0.729	<0.0001	2.099	0.679
FD_53_153	<0.0001	1.888	0.675	<0.0001	1.390	0.586
FD_94_194	<0.0001	2.979	0.76	<0.0001	2.006	0.668
FDH_33_133	<0.0001	2.345	0.725	<0.0001	1.716	0.634
FArea02	<0.0001	1.379	0.597	0.0025	1.108	0.521
FArea03	<0.0001	1.932	0.674	<0.0001	1.523	0.598
FR05_psu	<0.0001	0.692	0.598	<0.0001	0.705	0.6
FR06_psu	<0.0001	1.703	0.646	<0.0001	1.618	0.629
FR08_psu	<0.0001	1.758	0.652	<0.0001	1.703	0.642

OR: odd ratio, AUC: area under the receiver operating characteristic curve.

In the female groups (Table 3), the results in the Women-18-50 group showed 12 characteristics with significant differences between normal waist and VO, with 11 of these characteristics being highly significantly different. The strongest indicator of VO in the Women-18-50 group is FD_94_194 (p ≤ 0.0001, OR = 2.979, AUC = 0.76). In Women-51-80, 10 of 13 differences in the characteristics with p-values < 0.05 were highly significant. The best predictor of VO is FD_43_143 (p ≤ 0.0001, OR = 2.099, AUC = 0.679), which is in contrast to the results for the Women-18-50, Men-18-50, and Men-50-80 groups. Additionally, FD_94_194 (p ≤ 0.0001, OR = 2.006, AUC = 0.668) and FR08_psu (p ≤ 0.0001, OR = 1.703, AUC = 0.642) are helpful predictors of normal waist and VO discrimination in this group.

### Analysis of the predictive power of combined characteristics

**Figure 2:**
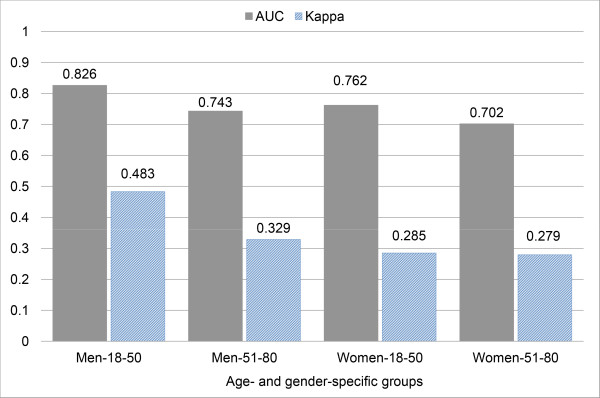
**A comparison of the predictive powers using the combined characteristics in 4 groups.** AUC: area under the receiver operating characteristic curve.

The diagnostic performances by AUC and Cohen’s kappa in the 4 age- and gender-specific groups are depicted in Figure 2. The AUC and kappa values of the prediction models in the 4 groups ranged from 0.826 to 0.702 and from 0.483 to 0.279, respectively. In comparing the single best indicator among the individual characteristics, the predictive powers of the prediction models improved marginally for the AUC values when we used the combined characteristics in all groups. For example, the AUC value of the best predictor in Women-51-80 is 0.679 for FD_43_143, whereas the AUC value of the combined characteristics is 0.702. Thus, the predictive power using the combined characteristics in Women-51-80 was improved by 0.023. The model constructed in this group included 8 characteristics (i.e., FDH_36_136, FDV_52_50, FDV_52_81, FDV_38_50, FD_53_153, FD_94_194, FDH_33_133, and FArea03 (FArea: area of the contour formed by the points)) and showed that the sensitivities for normal waist and VO were 0.542 and 0.734, respectively. Table 4 shows the detailed results for each prediction using combined characteristics. Table 5 lists the characteristics selected using the wrapper-based variable selection method in each group. The significant characteristics and selected characteristics identified by the variable selection method differed somewhat according to each model. In the age- and gender-specific models, several characteristics that showed a strong association with visceral obesity were included. FD_94_194, FArea03, and FR08_psu (FD(43,143) divided by FDV(52,50)) were included in the model for Men-18-50, and these characteristics had AUC values of 0.821, 0.704, and 0.694, respectively. FD_94_194 was included in the models for Men-51-80 and Women-51-80 and had AUC values of 0.735 and 0.668, respectively. The model for Women-18-50 included FD_43_143, FD_94_194, and FDH_33_133 and had AUC values of 0.729, 0.76, and 0.725, respectively.

**Table 4:** Detailed results of each prediction using combined characteristics

**Group**	**Class**	**Sensitivity**	**1-specificity**	**Precision**	**F-Measure**	**MCC**
Men-18-50	Normal	0.894	0.438	0.806	0.847	0.491
	VO	0.562	0.106	0.723	0.632	
Men-51-80	Normal	0.843	0.531	0.716	0.774	0.339
	VO	0.469	0.157	0.652	0.545	
Women-18-50	Normal	0.944	0.709	0.783	0.856	0.321
	VO	0.291	0.056	0.657	0.403	
Women-51-80	Normal	0.542	0.266	0.627	0.581	0.282
	VO	0.734	0.458	0.66	0.695	

VO: visceral obesity, MCC: Matthews correlation coefficient.

**Table 5:** Characteristics selected by wrapper-based variable subset selection in each group

**Group**	**N**	**Selected characteristics**
Men-18-50	8	FDV_52_50, FDV_52_81, FDV_138_50, FD_94_194, FArea03, FR05_psu, FR06_psu, FR08_psu
Men-51-80	6	FDV_52_50, FDV_81_50, FD_94_194, FR05_psu, FR06_psu, FR08_psu
Women-18-50	6	FDV_52_50, FD_43_143, FD_53_153, FD_94_194, FDH_33_133, FR08_psu
Women-51-80	8	FDH_36_136, FDV_52_50, FDV_52_81, FDV_38_50, FD_53_153, FD_94_194, FDH_33_133, FArea03

N: total number of features.

Our results indicate that the models constructed for the young and middle-age groups in the prediction of normal waist and VO for both the male and female groups are better than those in the elderly groups for both the use of individual and combined characteristics. In addition, the models built for the male groups in the same age groups are superior to those for the female groups for both the individual and combined characteristics.

## Discussion

Some studies have investigated the association of facial characteristics with diseases, obesity, or ethnic groups [13-29], but few studies have examined the direct association of VO with facial anthropometric characteristics. Levine and colleagues [16] hypothesized that facial cheek fat (as a facial characteristic) may increase in conjunction with visceral fat. By correlating the cheek area with VO using computed tomography (CT) and statistical analysis, they found a significant correlation of the cheek area with that of visceral fat and argued that buccal fat was likely to increase in patients along with visceral fat and that plump cheeks appear to be a risk factor for obesity-related metabolic complications. FArea03 (used in the present study) is a characteristic of cheek fat and chubby cheeks in the frontal face of subjects. Similar to the study by Levine and colleagues [16], our results indicated that FArea03 is significantly different between normal waist and VO in both men and women (p ≤ 0.0001, OR = 2.294, and AUC = 0.704 in Men-18-50; p ≤ 0.0001, OR = 1.850, and AUC = 0.662 in Men-51-80; p ≤ 0.0001, OR = 1.932, and AUC = 0.674 in Women-18-50; p ≤ 0.0001, OR = 1.523, and AUC = 0.598 in Women-51-80). This result indicates that VO subjects tend to have more cheek area than normal subjects. Furthermore, FD_43_143 (mandibular width in frontal face) and FD_94_194 (distance between both inferior ear lobes) are important indicators for discriminating between normal and VO subjects. Therefore, our results are consistent with the finding of an association between cheek fat and visceral fat, as demonstrated by Levine and colleagues [16]. In addition, in our previous study [14], Lee and colleagues examined the association between facial features and BMI and found that the classification of BMI status in women older than 40 years old was more difficult than that in women younger than 40 years old. The results of the present study are similar to the findings by Lee and colleagues [14]. We believe that the prediction of obesity in young and middle-aged individuals is more accurate than the prediction of obesity in elderly individuals due to the association of regional fat deposits in the face with both BMI and WC.

We observed facial characteristics in different age groups of the same gender in the differentiation of VO from normal waist. In comparing the male groups, the difference in FDV_38_50 was significant between the VO and normal subjects in the Men-18-50 group (p = 0.0378, OR = 1.116, AUC = 0.525), but this difference was not significant in the Men-51-80 group (p = 0.8675, OR = 1.006, AUC = 0.495). In the female groups, the FDV_52_50 and FDV_52_81 characteristics in the Women-18-50 group were not significantly different between normal waist and VO (p = 0.0885, OR = 1.077, AUC = 0.523 and p = 0.1246, OR = 0.934, AUC =0.515, respectively), but these results were significantly different in the Women-51-80 group (p = 0.0156, OR = 0.926, AUC = 0.526, p ≤ 0.0001, OR = 0.864, AUC = 0.544, respectively). Conversely, FDV_81_50 in Women-18-50 (p ≤ 0.0001, OR = 1.235, AUC = 0.558) was significantly different between normal waist and VO, but not in Women-51-80 (p = 0.1985, OR = 1.042, AUC = 0.507). We cannot explain the cause and effect relationships of these phenomenon because our study had a cross-sectional design; however, these particular characteristics highlight the subtle age differences by gender and should be helpful in distinguishing between VO and normal status in young and middle-aged individuals compared with elderly individuals in both males and females.

However, characteristics such as FD_43_143, FD_53_153, FD_94_194, FDH_33_133, FArea03, FR05_psu (FDH(33,133) divided by FD(43,143)), FR06_psu (FDH(33,133) divided by FDV(52,50)), and FR08_psu have a broad range of applicability for predicting normal waist and VO individuals because these characteristics had highly significant differences (p = <0.0001) in all groups and were commonly found in all age- and gender-specific groups. Among them, we believe that FR05, FR06, and FR08 are more important characteristics with broad applications, including medicine and forensics, because the image characteristics considered the ratio variables of two distances. We believe that the ratio variables of two distances in a facial image are less affected by the distortion that occurs at the time of photographing compared with general distances. Although the best predictors in the groups were FD_94_194 and FD_43_143, the ratio characteristics were significantly different enough to distinguish between the normal waist and VO individuals.

Generally, with variable selection that eliminates redundant or irrelevant characteristics, prediction models become more accurate, more cost-effective, and faster than those using full variable sets. After selection with the wrapper-based variable selection approach, the number of remaining features was small, but the diagnostic accuracy was slightly better than that of the model with full variable sets. In the experiments using the full set of characteristics without a variable selection approach, the AUC and kappa values, respectively, were 0.822 and 0.478 in Men-18-50, 0.737 and 0.307 in Men-51-80, 0.755 and 0.273 in Women-18-50, and 0.701 and 0.282 in Women-51-80. For instance, the AUC and kappa values in Women-18-50 were improved by 0.007 and 0.012, respectively, using the wrapper-based variable selection approach. In our experiments, after using the variable selection approach, the prediction accuracies slightly improved in all age- and gender-specific groups.

The results of the present study may provide clinical hints that enable alternative diagnosis of VO in the remote healthcare monitoring service, emergency medicine, and u-healthcare fields; the results may also lead to the development of advanced applications in predicting specific health problems.

## Conclusions

Visceral or abdominal obesity is a strong predictor for chronic disease, such as metabolic syndrome, diabetes mellitus, insulin resistance, and CVD, and human facial characteristics provide clinical indicators related to health complications, health problems, and obesity. In the present study, we tested the association of visceral obesity with facial anthropometric characteristics in adult Korean men and women and identified the best indicators for distinguishing between normal subjects and viscerally obese subjects using only simple facial characteristics with statistical analysis and data mining. We also compared the predictive powers of individual and combined characteristics in making better predictions.

The findings of the present study indicated that the distance between both inferior ear lobes was the strongest predictor of normal waist and VO in the young and middle-age groups and the elderly group for men as well as the young and middle-age groups for women, whereas the best indicator in the elderly female group was mandibular width. For both the individual and combined characteristics, the predictive values for the young and middle-age groups for both men and women were somewhat greater than those for the elderly groups. The models built for the male groups also appear to be superior to those built for women (in the same age groups) for both the individual and combined characteristics.

The present study is limited by ethnic differences because only data from Korean adults were analyzed in this experiment. In addition, drawing a cause and effect relationship between VO and facial characteristics is difficult because of the cross-sectional nature of this study. Finally, our results may have the potential to predict normal waist and VO using facial anthropometric characteristics. To the best of our knowledge, this report is the first to predict visceral obesity using individual and combined characteristics in age- and gender-specific groups.

## Competing interests

The authors declare that they have no competing interests.

## Authors’ contributions

BJL designed and developed the study and carried out the data analysis. BJL conducted all experiments, interpreted the results, and wrote the manuscript. JYK provided clinical information, participated in data analysis, and reviewed the results. Both authors read and approved the final manuscript.

## Pre-publication history

The pre-publication history for this paper can be accessed here:

http://www.biomedcentral.com/1472-6882/14/248/prepub
